# Effects of antibiotic resistance alleles on bacterial evolutionary responses to viral parasites

**DOI:** 10.1098/rsbl.2016.0064

**Published:** 2016-05

**Authors:** Flor I. Arias-Sánchez, Alex R. Hall

**Affiliations:** Institute of Integrative Biology, ETH Zürich, 8092 Zürich, Switzerland

**Keywords:** *Escherichia coli*, antibiotic resistance, experimental evolution, phage therapy

## Abstract

Antibiotic resistance has wide-ranging effects on bacterial phenotypes and evolution. However, the influence of antibiotic resistance on bacterial responses to parasitic viruses remains unclear, despite the ubiquity of such viruses in nature and current interest in therapeutic applications. We experimentally investigated this by exposing various *Escherichia coli* genotypes, including eight antibiotic-resistant genotypes and a mutator, to different viruses (lytic bacteriophages). Across 960 populations, we measured changes in population density and sensitivity to viruses, and tested whether variation among bacterial genotypes was explained by their relative growth in the absence of parasites, or mutation rate towards phage resistance measured by fluctuation tests for each phage. We found that antibiotic resistance had relatively weak effects on adaptation to phages, although some antibiotic-resistance alleles impeded the evolution of resistance to phages via growth costs. By contrast, a mutator allele, often found in antibiotic-resistant lineages in pathogenic populations, had a relatively large positive effect on phage-resistance evolution and population density under parasitism. This suggests costs of antibiotic resistance may modify the outcome of phage therapy against pathogenic populations previously exposed to antibiotics, but the effects of any co-occurring mutator alleles are likely to be stronger.

## 1. Background

The evolution of resistance to antibiotics impedes treatment of infections [1]. Lytic bacteriophages, which are abundant in nature and clinically relevant environments including the human gut [2], are regarded as a promising alternative or adjunct [3,4]. Despite the central role of phages in bacterial ecology and evolution [5], the interplay between antibiotic resistance and bacterial responses to phages remains poorly understood. If antibiotic-resistance alleles alter bacterial sensitivity or adaptation to phages, then the success of phage therapy against a given infection will depend strongly upon which antibiotics the pathogen population has been exposed to previously.

Antibiotic resistance could modify responses to phages by altering either sensitivity to phage infection or the rate of phage-resistance evolution. Altered sensitivity to phages might result, for example, from wide-ranging pleiotropic changes in gene expression caused by some antibiotic-resistance alleles [6]. These pleiotropic effects could also influence rates of phage-resistance evolution, if they alter the frequencies or fitness effects of phage-resistance mutations. Even if this is not the case, antibiotic resistance is frequently associated with reduced bacterial population growth [7], potentially inhibiting phage-resistance evolution by reducing mutation supply. We therefore hypothesized that both population growth in the presence of phages and the frequency of resistance evolution would vary among bacteria with different antibiotic-resistance alleles.

We tested this by quantifying the effect of resistance mechanisms against several antibiotics, encoded by either chromosomal mutations or plasmids, on bacterial growth and resistance evolution in phage treatments. We also included a bacterial mutator genotype with defective DNA mismatch repair, with the rationale that drug resistance and elevated mutation rates are frequently associated in clinical scenarios [8].

## 2. Material and methods

### (a) Organisms and culture conditions

We used *Escherichia coli* K12-MG1655 (wild-type or ‘WT’ hereafter) and nine genotypes derived from it (table 1). *Escherichia coli* is a relevant model system given its clinical importance [9], increasing antibiotic resistance [1] and potential as a target for phage therapy [10]. We used tailed bacteriophages from each *Caudovirales* family: T4 (*Myoviridae*), T7 (*Podoviridae*), and a lytic version [11] of phage *λ* (*Siphoviridae*). Experiments were performed at 37°C in Luria–Bertani medium supplemented with 10 mM MgSO_4_ and 10 mM Tris HCl.

**Table 1.** Bacterial genotypes. Information about their origins is included in the electronic supplementary material. Kan, kanamycin; Cipro, ciprofloxacin; Rif, rifampicin; Strep, streptomycin; Sulf, sulfonamide; Tet, tetracycline; Amp, ampicillin.

genotype name	gene mutated	antibiotic resistance
WT	—	‘sensitive’
MUT	*mutS*	Kan
D87G	*gyrA*	Cipro
S83L	*gyrA*	Cipro
D516G	*rpoB*	Rif
S512F	*rpoB*	Rif
K43N	*rpsL*	Strep
K88R	*rpsL*	Strep
RSF1010	plasmid	Strep + Sulf
RP4	plasmid	Tet + Amp + Kan

### (b) Bacterial population growth

We performed a randomized fully factorial (4 phage treatments × 10 bacterial genotypes) experiment with 24 independent populations in each combination (*n* = 960). We added approximately 10^8^ plaque forming units (PFU) of phage (T7, T4 or *λ*), or sterile medium in the phage-free treatment, when bacterial populations were growing exponentially (mean optical density at 600 nm of 0.130). To measure biomass in each culture, we recorded optical density upon phage introduction (0 h) and, based on previous studies [12,13], after 20 h and 72 h using an M2 Spectramax spectrophotometer (Molecular Devices, Sunnyvale, CA, USA), correcting values by the score for sterile medium.

### (c) Changes in phage sensitivity

At the end of the experiment, we plated population aliquots on agar (1.5%) with and without approximately 10^10^ PFU of phage spread over the surface. We took inhibition of colony formation as an indication of sensitivity to phage, scoring each population for viable colony growth in the presence and absence of each phage after 24 h. We scored populations that did not form viable colonies on phage-free plates as extinct.

### (d) Mutation rates

We estimated mutation rate (*μ*) per genome per replication to phage resistance for every bacterial genotype × phage combination by fluctuation assays [14] (electronic supplementary material) using the MSS-MLE method in falcor [15]. Because changes in mutation rate resulting from antibiotic-resistance alleles or mutator alleles may vary across genes or phenotypes [16], we tested each bacteria × phage combination separately.

## 3. Results

### (a) Final bacterial density varies among genotypes and phage treatments

**Figure 1.**
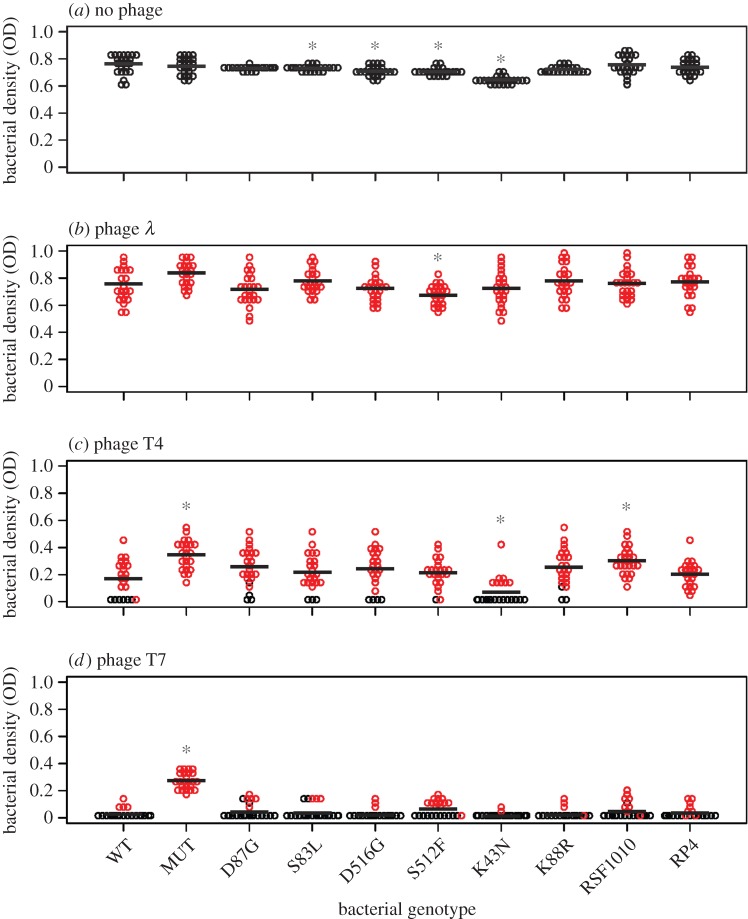
Bacterial population densities after 72 h of phage exposure. Each circle represents a single population. Red denotes populations that formed viable colonies upon plating on agar supplemented with the phage they were exposed to. Phage sensitivity for populations from the phage-free treatment is given in the electronic supplementary material, table S1.

On average, bacterial population densities at the end of the experiment were lowest after exposure to phages T4 and T7 (figure 1). Accordingly, the number of extinct bacterial populations was greatest with T4 and T7 (*λ* = 0, T4 = 37, T7 = 164). For phage *λ*, despite a clear effect over the first 20 h (electronic supplementary material, figure S1), final bacterial densities were similar on average to the phage-free control. Across phage treatments, final population density varied among bacterial genotypes (*p* < 0.001 in each treatment by one-way ANOVA or non-parametric alternative; further details of analyses are given in the electronic supplementary material). Pairwise comparisons showed that in most cases (21/24), antibiotic-resistance alleles did not significantly alter population density relative to the WT after exposure to phages (figure 1). The strongest effect was for the streptomycin-resistant genotype K43N with phage T4 (final OD = 0.11 on average, compared with 0.22 for the WT). K43N also reached the lowest average population density in the absence of phages (figure 1*a*), and frequently went extinct with both T4 (67%) and T7 (92%). By contrast, the mutator genotype consistently attained higher population sizes than the WT after exposure to the most effective phages (T4: *p* < 0.0001; T7: *p* < 0.0001), but not in the absence of phages. These effects were relatively strong (final OD was 1.8-fold and 4.6-fold higher on average than the WT) compared with those for antibiotic-resistance alleles, and the mutator was the only genotype with no extinctions in any phage treatment.

### (b) Surviving populations frequently show reduced sensitivity to phages

Every population exposed to phage *λ* produced viable colonies on agar plates containing this phage (figure 1*b*). Populations from the phage-free treatment also frequently produced colonies on *λ* plates (electronic supplementary material, table S1), but the fraction of populations forming colonies was less than in phage-exposed populations (one-sample *t*_9_ = 4.46, *p* = 0.002). Similarly, for phages T4 and T7 the fraction of populations forming colonies on phage-agar was higher among phage-exposed populations than controls (figure 1*c,d*; electronic supplementary material, table S1; T4: paired *t*_9_ = 9.35, *p* < 0.0001; T7: no viable colonies among non-mutator control populations). In these treatments, variation of the fraction of populations producing colonies on phage-plates was correlated with variation of average final population density across bacterial genotypes (T4: *r*^2^ = 0.67, *p* = 0.004; T7: *r*^2^ = 0.92, *p* < 0.0001; electronic supplementary material, figure S2). Thus, acquisition of reduced phage sensitivity was associated with increased population growth and varied among bacterial genotypes in the T4 and T7 treatments.

### (c) What drives variation among bacterial genotypes?

Variation of rates of adaptation to phage treatments among bacterial genotypes could stem from differences in intrinsic population growth in our experimental environment (*N*, measured in the absence of phages) or mutation rate towards phage resistance (*μ*, measured by fluctuation tests), which together determine the supply rate of phage-resistance mutations.

In the phage T4 treatment, variation of *N* among bacterial genotypes was correlated with variation of both their final population density in the presence of phage (*r*^2^ = 0.44, *p* = 0.04) and the proportion of populations that formed viable colonies on phage-agar plates (*r*^2^ = 0.50, *p* = 0.02). However, both of these associations were driven by bacterial genotype K43N (*r*^2^ = 0.02, *p* = 0.75; *r*^2^ = 0.03, *p* = 0.66 in analysis without K43N), which showed the lowest population growth in the absence of phages (figure 1*a*) and a relatively infrequent capacity to form colonies on T4-agar after exposure to the phage (figure 1*c*). For the other phage treatments, *N* in the absence of phages was not strongly correlated with final population density (T7: *r*^2^ = 0.12, *p* = 0.35; *λ*: *r*^2^ = 0.24, *p* = 0.15) or proportion of populations forming viable colonies on phage-supplemented agar (T7: *r*^2^ = 0.02, *p* = 0.74, *λ*: no variation in phage sensitivity).

**Figure 2.**
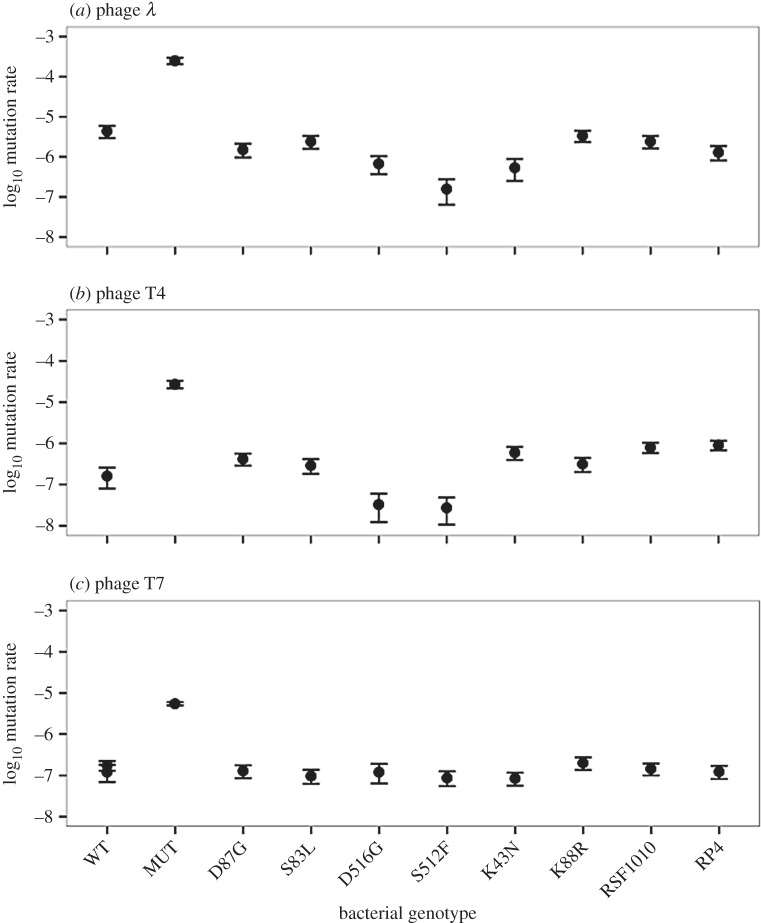
(*a*–*c*) Mutation rates to phage resistance. Error bars represent 95% CIs. Note that MUT in the T7 treatment was assayed in a separate block alongside independent controls (WT); the value for WT was similar in both blocks (−6.92 and −6.76).

Unsurprisingly, mutation rate towards phage resistance was consistently highest for the mutator (figure 2). With T4 and T7, where the mutator reached higher population densities than other genotypes, this generated a positive association between mutation rate and average final population density (T4: *r*^2^ = 0.31, *p* < 0.01, T7: *r*^2^ = 0.96, *p* = 0.0001; same analysis excluding the mutator: T4: *r*^2^ = 0.0003, *p* = 0.96, T7: *r*^2^ = 0.04, *p* = 0.59). Consistent with the advantage of high mutation rate resulting from accelerated phage-resistance evolution, mutation rate was also correlated with the frequency of populations forming viable colonies on phage-agar at the end of the experiment in our T7 treatment (*r*^2^ = 0.82, *p* < 0.001; *r*^2^ = 0.03, *p* = 0.64 excluding the mutator). With T4, where mutator advantage was weaker than with T7 and other bacterial genotypes frequently evolved reduced sensitivity to this phage, mutation rate did not predict changes in phage sensitivity on agar (*r*^2^ = 0.06, *p* = 0.50). There was also a positive association between *μ* and average final OD with phage *λ* (*r*^2^ = 0.46, *p* = 0.03), with average OD being highest for the mutator and lowest for the rifampicin-resistant genotype S512F, which had the highest and lowest mutation rates towards *λ*-resistance, respectively (figure 2*a*), consistent with mutation rate contributing to adaptation here even though all exposed populations formed viable colonies on *λ*-agar.

## 4. Discussion

Contrary to our expectation that pleiotropic effects of antibiotic resistance would alter responses to lytic phages, we found only weak effects on growth and adaptation to phage treatments. The strongest effect for an antibiotic-resistance allele (K43N with phage T4) was linked to a relatively large growth cost in the absence of phages that was in turn associated with relatively infrequent resistance evolution against some phages. Other studies have found similar growth costs (5–15% reduction in biomass production) for the resistance mechanisms in our experiment (electronic supplementary material) and for other types of resistance in different species [7], indicating costs of antibiotic resistance may constrain adaptation to phages in other scenarios. This was not the case for the mutator, which was highly beneficial in phage treatments, probably because it increased the frequency of phage-resistance mutations. Such hypermutable genotypes are frequently found among clinical isolates [17] and often associated with antibiotic resistance [8].

Why did the effects of mutation rate and costs of resistance vary among phage treatments? Both factors influence total mutation supply rate (*N* × *μ*), but this does not always translate to variation in rates of resistance evolution among genotypes. The mutator allele had the largest effect on survival and adaptation in our T7 treatment, where other genotypes frequently went extinct. Consistent with recent multi-phage experiments [18], this suggests mutator advantage is the strongest when phage-resistance mutations are highly beneficial and non-mutator genotypes evolve resistance relatively infrequently. By contrast, the effect of the cost of antibiotic resistance on adaptation for genotype K43N was the strongest with phage T4. We speculate that this effect was reduced in the *λ* and T7 treatments, because there was little variability in resistance to these phages among non-mutator genotypes (where all or very few populations evolved resistance) so the reduced mutation supply rate of K43N had no effect on adaptation. The variation among these phages in terms of their average impact on bacterial population density is consistent with previous observations of differences in their life-history traits [19].

The pleiotropic effects of antibiotic resistance on phage sensitivity and adaptation were smaller than expected, but mutator alleles had relatively large effects. This suggests the outcomes of phage therapy against drug-resistant infections and the distribution of antibiotic resistance in phage-parasitized populations in nature may depend both on costs of antibiotic resistance and on whether or not mutator alleles co-occur on the same genome. Our observations were limited to a single mutator genotype and species, and are likely strongly influenced by the short-term effects of phage-resistance evolution, driven in many cases by mutations occurring prior to phage exposure. Nevertheless, previous work suggests that hypermutability is also advantageous during longer-term coevolution [20], often due in other species to the same mechanism as here [17], and frequently co-occurs with antibiotic resistance [8,21].

## Supplementary Material

Supplementary Material

## Data accessibility

Datasets available at Dryad: http://dx.doi.org/10.5061/dryad.90qb7.
