# Correction to ‘Effects of antibiotic resistance alleles on bacterial evolutionary responses to viral parasites’

**DOI:** 10.1098/rsbl.2016.0759

**Published:** 2016-10

**Authors:** Flor I. Arias-Sánchez, Alex R. Hall

*Biol. Lett.* **12**, 20160064. (Published online 18 May 2016) (doi:10.1098/rsbl.2016.0064)

After publication of our article, we found our estimates of population sizes used to infer mutation rates in Figure 2 had been miscalculated and the correct values for figure 2*a*,*b* and part of 2*c* are lower than those shown in the published article. Our conclusions from this section (that the mutator had the highest estimate for all three phages) are largely unchanged, however, the second sentence of §3c paragraph 3 in the Results section should now read: ‘With T7, where the mutator reached higher densities than other genotypes, this generated a positive association between mutation rate and average final population density (*r*^2^ = 0.90, *p* < 0.0001; *r*^2^ = 0.04, *p* = 0.59 excluding the mutator); for T4 the association was positive but not significant (*r*^2^ = 0.31, *p* = 0.09; *r*^2^ = 0.001, *p* = 0.93 excluding the mutator)’. A corrected version of figure 2 is included and the Dryad entry has been updated here doi:10.5061/dryad.90qb7.3.

**Figure 2.**
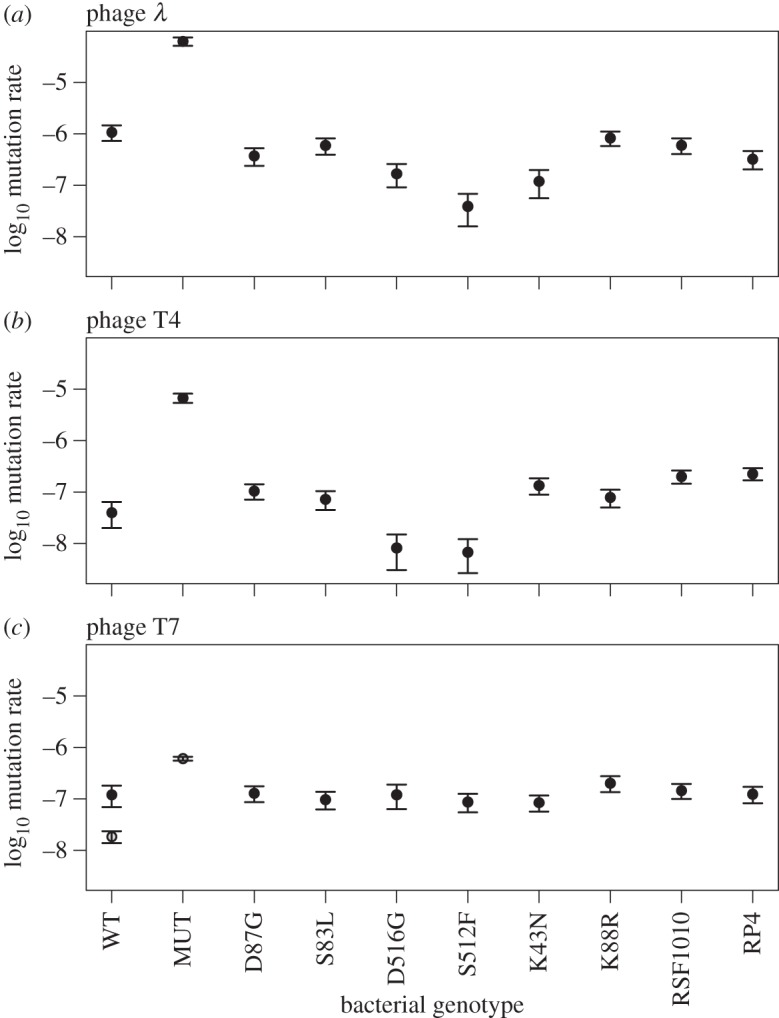
(*a*–*c*) Mutation rates to phage resistance. Error bars show 95% CIs. Note that MUT in the T7 treatment was assayed in a separate block alongside independent controls (WT, for which we obtained a lower estimate in this block than in the previous block: −7.73 compared to −6.92; this suggests we may be underestimating MUT relative to other genotypes in this treatment; nevertheless, as in the other phage treatments MUT had the highest estimate of all genotypes).

