# Supplementary material for: Effects of antibiotic resistance alleles on bacterial evolutionary responses to viral parasites
Source: Biol Lett. 2016 May;12(5):20160064. doi: 10.1098/rsbl.2016.0064 (PMC4892242; doi:10.1098/rsbl.2016.0064)
Supplement: Supplementary Material [file rsbl20160064supp1.docx]

**Supplementary Material**

**Origin of bacterial genotypes**

Antibiotic resistance alleles were identified in previous studies following selective plating and sequencing (D516G and S512F - Hall 2013; D87G and S83L - Tazzyman & Hall 2015; K43N and K88R - Angst & Hall 2013), or insertion of plasmids obtained from the Leibniz Institut DSMZ-Deutsche Sammlung von Mikroorganismen und Zellkulturen (Angst & Hall 2013). All of these alleles have also been associated with antibiotic resistance in earlier studies by other researchers (Reynolds 2000; Garibyan 2003; Trindade 2009). The mutator genotype has a kanamycin-resistance gene (*kan*) that was used as a selective marker during construction of this strain.

The relative growth capacities of all of these genotypes have been previously measured by pairwise competitions in the same growth medium (Trindade *et al*. 2009; Silva *et al*. 2011; Angst & Hall 2013; Tazzyman & Hall 2014). These studies agree qualitatively with the relative growth of different genotypes in our phage-free control treatment: K43N is the most costly allele, while others had small or negligible fitness effects, and the mutator allele is approximately neutral over this timescale.

**Fluctuation assays**

From diluted overnight cultures of each genotype, we transferred 30-300 cells (*N*_0_) into sterile LB medium to inoculate replicate independent populations (20 per genotype per fluctuation assay). Once populations reached stationary phase (after 20h at 37°C), we determined the number of viable cells (*N*_t_) by dilution and plating on LB-agar (3-6 replicates per fluctuation assay). We estimated the number of phage resistant mutants (*r*) by plating a sample (λ and T4) or the entire population (T7) onto LB-agar (1%) containing a sufficient phage concentration that only resistant mutants can grow.

To determine whether colonies growing on the selective plates we used in our fluctuation assays were likely to have acquired heritable resistance to the relevant phage, we picked colonies for each type of selective plate and tested them for phage resistance. For λ and T4, selective plates were prepared exactly as in the fluctuation tests reported in the main text, although on a different day, and 18 replicate populations of the wild-type were plated onto these selective plates before overnight incubation. For T7, we used a slightly modified protocol, using a pin-replicator to plate 96 populations of the wild type onto a square agar plate at the same phage concentration, in order to obtain enough wild-type colonies (as shown in Fig. 1, colonies for the wild-type are less common here than with the other phages). After overnight incubation, a single colony was randomly chosen from each plate (18 colonies per phage, each derived from a different population). We then restreaked colonies on phage-free agar to purify them of phages carried over from the selective plate, before streaking them across a line of the relevant phage that had been dried onto an LB-agar plate. Alongside these colonies, we streaked colonies of the wild type that had never been exposed to phages. Following previous authors (Buckling et al. 2002; Brockhurst et al. 2007), we then scored colonies as resistant if there was no visible inhibition of growth by phages after overnight incubation. For 17/18 (λ), 18/18 (T4) and 17/18 (T7) colonies from selective plates, there was no inhibition by phages. By contrast, every control colony was susceptible to phages on the same plates.

**Statistical Analyses**

We tested for variation of final population density among bacterial genotypes in each phage treatment by one-way ANOVA, before comparing each genotype against the wild type using pairwise *t*-tests with Dunnet’s method for multiple comparisons against one control (Dunnet, 1955). We used a Wilcoxon test and a non-parametric equivalent for multiple comparisons (Steel, 1959) in the phage-free and T7 treatments due to heteroscedasticity (Levene’s tests: *P*<0.0001). To determine whether genotypes with relatively high average population densities at the end of the experiment were also more likely to form viable colonies on phage-supplemented agar, we first estimated, for each genotype in each phage treatment, the proportion (*Pr*) of populations that formed viable colonies on phage-agar and the final average OD ($\bar{OD}$). We then tested whether variation of *Pr* predicted variation of $\bar{OD}$ among bacterial genotypes in each phage treatment. We performed similar regression analyses to test for associations between mutation rate toward phage resistance or growth in the absence of phages and $\bar{OD}$ or *Pr*.

**Table S1.** Percentage of 24 control populations that were never exposed to phages, which formed viable colonies on phage-supplemented agar.

|  | Proportion of populations forming colonies (%) | | |
| --- | --- | --- | --- |
|  | λ | T4 | T7 |
| WT | 75 | 21 | 0 |
| MUT | 100 | 71 | 8 |
| D87G | 58 | 8 | 0 |
| S83L | 79 | 17 | 0 |
| D516G | 25 | 0 | 0 |
| S512F | 75 | 17 | 0 |
| K43N | 17 | 0 | 0 |
| K88R | 75 | 0 | 0 |
| RSF1010 | 54 | 8 | 0 |
| RP4 | 79 | 17 | 0 |

**Figure S1.** Population densities after 20h of phage exposure. Panels represent phage treatments and columns bacterial genotypes. Each circle represents a single population.

**Figure S2.** Average population density for each bacterial genotype (separate circles) as a function of the proportion of populations forming colonies on phage-supplemented agar. The λ treatment is not shown because all populations formed colonies here. The blue line shows the fit of a linear regression in each treatment

**References**

Angst, D. & Hall, A.R. 2013 The cost of antibiotic resistance depends on evolutionary history in Escherichia coli. BMC Evol. Biol. **13**: 163.

Brockhurst, M.A., Morgan, A.D., Fenton, A. & Buckling A 2007 Experimental coevolution with bacteria and phage: The Pseudomonas fluorescens - Φ2 model system. Infection Genetics and Evolution **7**: 547-552.

Buckling, A., Rainey, P.B. 2002 The role of parasites in sympatric and allopatric host diversification. Nature **420**: 496-499.

Dunnett, C.W. 1955 A multiple comparison procedure for comparing several treatment with a control. J. Am. Stat. Assoc. **50**, 1096-1121.

Garibyan, L., Huang, T., Kim, M., Wolff, E., Nguyen, A., Nguyen, T., Diep, A., Hu, K.B., Iverson, A., Yang, H.J. & Miller, J.H. 2003 Use of the rpoB gene to determine the specificity of base substitution mutations on the Escherichia coli chromosome. DNA Repair **2**: 593-608.

Hall, A.R. 2013 Genotype-by-environment interactions due to adaptation and antibiotic resistance in Escherichia coli. J. Evol. Biol. **26**: 1655-1664.

Reynolds, M.G. 2000 Compensatory evolution in rifampin-resistant Escherichia coli. Genetics **156**: 1471-1481.

Steel, R. G. D. 1959 A multiple comparison rank sum. *Biometrics* **15**, 560–572.

Tazzyman, S.J. & Hall, A.R. 2015 Lytic phages obscure the cost of antibiotic resistance in Escherichia coli. The ISME journal **9**: 809-820.

Trindade, S., Sousa, A., Xavier, K.B., Dionisio, F., Ferreira, M.G., Gordo, I. 2009 Positive epistasis drives the acquisition of multidrug resistance. PLoS Genet. **5**: e1000578.
